# Editorial

**Published:** 1882-06

**Authors:** 


### Editorial.

The April issue of this journal contained a statement, in the letter of our New York correspondent, which requires correction. It is there said that the New York Obstetrical Society had voted to give its reports to the *New York Medical Journal*, in consequence of the unpopularity of the editor of the *Journal of Obstetrics* with the New York gynæcologists. Had our attention been sooner directed to this personal statement, it should have been corrected at an earlier date.

The facts of the case, as learned by us, are that the New York Obstetrical Society offered its transactions to the *Journal of Obstetrics* under certain conditions of a business nature, which the publishers of that journal were unwilling to accept. The publishers of the *New York Medical Journal*, being, of course, anxious to secure for their columns such valuable material, did accept the conditions under which the transactions had been offered to the other journal named, and thus the change was effected. There was in the entire matter, from first to last, no element whatever of personal feeling, the entire proceeding being conducted and decided on business principles.

Though late in making this correction, it is none the less necessary, both for the reputation of Dr. Mundé and of ourselves. The former is not only a brother medical editor who is as highly esteemed by us as any single member of that guild, but is a gentleman whose reputation as a specialist is as wide as this continent. No man could sit down to write the list of eminent American gynæcologists and obstetricians, without including his name, and if the writer were specially well informed, Dr. Mundé's name would appear very high on the list. The latter has in no sense lost in popularity with the New York obstetricians and gynæcologists; and if he had, it would be no reflection upon him. We have taken the trouble to get at the facts in this case, and feel that the reparation which we desire to make in this connection is simply an opportunity to pay a well-deserved compliment to a distinguished American physician.

---

THE following item in regard to Samuel Piercy, the actor, who died of small-pox some time ago, is copied from the New York correspondence of the *Philadelphia Press*: "He was one of a half-dozen intelligent men I ever knew to be influenced by the crazy howls of the anti-vaccination fanatics. Jebb and Bergh, and the rest of the mistaken lot, had managed to convince him that the risks lurking in the preventive were worse than the dangers of the disease. Before leaving New York, a few weeks ago, he laughingly rejected the advice of friends who urged him to be vaccinated. He was a convert to the views of Jebb and Bergh, and he paid the penalty of martyrdom."—*Michigan Med. News.*

---

PESSARY RETAINED FOR ELEVEN YEARS.—I read in this month's *Courier* the report of a case by J. G. Earnest, in which a pessary was retained in the vagina for six years. I can nearly discount that. One year ago, Mrs. H——, of Chicago, while on a visit to Charlotte, where I was then engaged in practice in company with Dr. L. A. Lebeau, formerly of St. Louis, called upon my associate, and consulted him in reference to a persistent leucorrhœa and stricture of the vagina. Upon examination, he discovered firmly imbedded in the folds of the vagina, and covered nearly its entire circumference in a mass of granulations and cicatricial tissue, a hard rubber ring pessary, which she informed the doctor had been placed there eleven years before by a physician of Chicago, and had remained there ever since, having never been once removed.—*Cour. of Med.*

